# Peroxisomal ether-glycerophospholipid synthesis is dysregulated after TBI

**DOI:** 10.1016/j.jlr.2025.100821

**Published:** 2025-05-07

**Authors:** Amir A. Mehrabani-Tabari, Nivedita Hegdekar, Sabrina Bustos, Yulemni Morel, Yuanyuan Ji, Sazia Arefin Kachi, Olivia Pettyjohn-Robin, Sagarina Thapa, Maya Bhattiprolu, Marta M. Lipinski, Jace W. Jones, Chinmoy Sarkar

**Affiliations:** 1 Shock, Trauma and Anesthesiology Research (STAR) Center, Department of Anesthesiology, University of Maryland School of Medicine, Baltimore, MD, USA; 2 Department of Pharmaceutical Sciences, University of Maryland School of Pharmacy, Baltimore, MD, USA; 3 Department of Biology, University of Maryland, College Park, MD, USA; 4 Department of Anatomy and Neurobiology, University of Maryland School of Medicine, Baltimore, MD, USA

**Keywords:** traumatic brain injury (TBI), lipid, ether-glycerophospholipids, neurodegeneration

## Abstract

Ether-glycerophospholipids (ether-GPs), the ether bond- (– O –) containing glycerophospholipids, are major components of the brain lipidome. Ether-GPs play a crucial role in regulating neuronal function, and their deficiency has been implicated in many neurodegenerative diseases. However, how they are affected after traumatic brain injury (TBI) is not known. Our data demonstrate a significant decrease in ether-GPs abundance in the mouse cortex following controlled cortical impact (CCI)-induced TBI. This is at least in part due to the impairment of peroxisomal ether-GP synthesis in the mouse brain after TBI. We detected dysregulation of peroxisomal ether-GPs synthesizing enzymes – glyceronephosphate-O-acyltransferase (GNPAT) and alkylglycerone phosphate synthase (AGPS) in the injured mouse brains. Our data demonstrate a significant decline in GNPAT level in the peroxisomal fraction and a marked accumulation of AGPS in the cytosol of mouse cortices after TBI. To restore the ether-GP level in the injured brain, we treated TBI mice with an ether-GP precursor, 1-O-octadecylglycerol (OAG), to bypass the peroxisomal ether-GPs synthesizing steps. OAG partially restored the levels of several ether-GPs, attenuated inflammatory cytokine expression, and improved their functional recovery after TBI. Taken together, our data demonstrate that the decline in ether-GPs abundance after TBI is at least in part due to the impairment in peroxisomal ether-GPs synthesis and that restoration of ether-GPs by OAG treatment can improve TBI outcomes.

Ether-glycerophospholipids (ether-GPs) are a type of glycerophospholipid that contain an ether bond (– O –) that binds an aliphatic chain to their glycerol backbone (1, 2) (supplemental Fig. S1). Generally, two hydroxyl groups at *sn-1* and *sn-2* positions of the glycerol moiety of glycerophospholipids remain attached to fatty acyl chains through ester bonds (3). These types of glycerophospholipids are called diacyl-glycerophospholipids. Ether-GPs also have a similar structure, except that they remain attached with an alkyl chain through an ether bond instead of an ester bond at the *sn-1* position of their glycerol backbone. There are two types of ether-GPs based on the presence or absence of unsaturation next to the ether bond in the alkyl chain attached to it. One is called alkyl-ether-GPs which lacks the unsaturation, and the other one that contains the unsaturation next to the ether bond is called alkenyl or vinyl ether-GPs or plasmalogens (1, 2, 3, 4, 5).

Ether-GPs constitute almost 20% of total phospholipids (1, 5). They are the major structural components of the cellular membrane. They allow tighter packing of membrane lipids and, consequently, provide structural rigidity to the membranes. Ether-GPs are important constituents of membrane lipid rafts, which serve as docking platforms for various signaling molecules. Their roles have been implicated in ERK and Akt signaling and many other pathways. They also act as endogenous receptors for peroxisome proliferator-activated receptor γ (PPARγ) (1, 4). They play a crucial role in membrane fusion and trafficking by their ability to induce a negative hexagonal membrane structure (1). Vinyl (alkenyl)- ether-GPs are known to protect other membrane components by scavenging reactive oxygen species (ROS) as sacrificial antioxidants (1).

Ether-GPs are synthesized in a concerted way by peroxisomes and endoplasmic reticulum (ER) (1, 4, 5, 6, 7). The synthesis begins within peroxisomes by the sequential activities of peroxisomal enzymes glyceronephosphate-O-acyltransferase (GNPAT) and alkylglycerone phosphate synthetase (AGPS) which generate ether-GPs precursor 1-O-alkyl-glycerol phosphate, which is then transported and converted to fully formed ether-GPs in the ER (1, 4, 5, 6, 7). Any defects in peroxisomal ether-GPs synthesis disrupt cellular ether-GPs homeostasis which can adversely affect cellular functions.

Ether-GPs are highly abundant in the brain (1, 5). They are one of the major constituents of myelin. They stabilize myelin structure and protect it from oxidative damage (5, 6, 7). They are also enriched in synaptic vesicles and play an important role in neurotransmitter release in the presynaptic cleft. As a result, ether-GPs dysregulation adversely affects neuronal function (1, 7, 8, 9). Defects in peroxisomal ether-GPs synthesis due to the mutations in peroxisomal enzymes or its biogenesis factors in genetic disorders, such as rhizolemic chondrodysplasia punctata, severely affect central nervous system development and functions causing microcephaly, reduced head growth, intellectual disability, epileptic seizures and motor impairment (1, 4, 6, 7). Decrease in Ether-GPs abundance has also been detected in different neurodegenerative diseases including Alzheimer’s disease (AD) and Parkinson’s disease (PD) (1, 4, 7, 10, 11). Reduced levels of plasmalogen have been detected in the cerebral and cerebellar white and gray matter and in the plasma of patients with AD. This might be caused by Aβ-mediated peroxisomal dysfunction and decrease in AGPS (1, 10). Plasmalogen decrease has also been reported in Lewy body dementia (4, 12). Similarly, reduced level of plasmalogen has been detected in the lipid raft isolated from post-mortem frontal cortex and plasma and erythrocytes of patients with PD (4, 13). Ether-GPs decline has been also reported in psychotic disorders including schizophrenia (1, 14, 15), and in neurodevelopmental disorders such as Rett syndrome, autism, and attention-deficit hyperactivity disorder (ADHD) (4, 16).

Although ether-GPs deficiency has been well-documented in different neurological disorders, how ether-GPs are affected in response to traumatic brain injury (TBI), an injury-inflicted acute and progressive neurodegenerative disease is not known. TBI is caused by the physical impact of external forces to the head due to falls, accidents, assaults, combat-related blast exposures or sporting activities (17, 18). In the US, more than 200,000 TBI-related hospitalizations were reported in 2020 (https://www.cdc.gov/traumaticbraininjury/pubs/tbi_report_to_congress.html). It is one of the major causes of death among people of all ages and those who survive the initial impact of TBI may develop life-long neurological impairments including motor and cognitive dysfunctions. In TBI, the primary mechanical injury to the brain triggers activation of biochemical events leading to both acute and progressive neurodegeneration and neuroinflammation. The biochemical changes include glutamate excitotoxicity, disruption of calcium balance, oxidative stress, endoplasmic reticulum stress, organellar dysfunction, and perturbation of lipid homeostasis (17, 18, 19, 20). However, the role and function of ether-GPs in the pathophysiology of TBI has not been studied before.

In this study, we demonstrate that ether-GPs levels are downregulated in the mouse cortical tissue due to the impairment of peroxisomal ether-GPs synthesis following controlled cortical impact (CCI)-induced moderate TBI. Our data also indicates that restoration of ether-GPs levels by supplementation with an ether-GP precursor able to bypass peroxisomal dysfunction has beneficial effect on TBI outcomes.

## Materials and Methods

### Controlled cortical impact

All surgical procedures and animal experiments were performed according to the protocols approved by the Animal Care and Use Committee of the University of Maryland and as per the guidelines and regulations of the Animal Care and Use Committee of the University of Maryland. Moderate TBI was induced in male *C57BL6/J* mice (20–25 g) under surgical anesthesia (2%–3% isoflurane evaporated in a gas mixture containing 70% N_2_O and 30% O_2_) by controlled cortical impact (CCI) using a custom-made microprocessor-controlled and compressed air-driven pneumatic impactor of 3.5-mm diameter tip with an impact velocity of 6 m/s and a deformation depth of 2 mm following a 4-mm craniotomy on their central aspect of the left parietal bone as described previously (21). Since in TBI experiments males and females cannot be combined as the injury outcomes and severity are affected by hormones and body weights differences of male and female, we chose male mice because clinically men are affected by TBI to a much higher extent than women (including higher numbers of emergency room visits, rates of hospitalization, and of death) (https://www.cdc.gov/traumaticbraininjury/pubs/tbi_report_to_congress.html).

### OAG supplementation

Mice (sham and TBI) were fed with chow (18% protein diet, cat # 2018SX, Envigo) supplemented with 2% (w/w) 1-O-Octadecyl-rac-glycerol (OAG, cat # 4014011, Bachem) starting from 7 days prior to TBI till the end of the experiments (PID 1, 3 or 28). This custom-made OAG-supplemented chow was prepared by Envigo. Control mice were fed with the same chow without OAG.

### Lipidomic analysis

#### Materials

LC-MS grade acetonitrile (ACN), methanol (MeOH), water (H_2_O), and *n*-propanol were purchased from Fisher Scientific. HPLC grade tert-Butyl methyl ether (MTBE), chloroform, ammonium formate, and formic acid were purchased from Sigma Aldrich. EquiSPLASH lipidomix was purchased from Avanti Polar Lipids, Inc. An additional five fatty acid internal standards including docosahexaenoic acid-d_5_, eicosapentaenoic acid-d_5_, arachidonic acid-d_11_, palmitic acid-d_9_ and *α*-linolenic acid-d_5_ were purchased from Cayman Chemical. An additional four ceramides including C13 galactosyl(*β*) Ceramide-d_7_ (d18:1-d_7_/13:0), C15 glucosyl(*β*) Ceramide-d_7_ (d18:1-d_7_/15:0), C15 lactosyl(*β*) Ceramide-d_7_ (d18:1-d_7_/15:0), and C13-dihydroceramide-d_7_(d18:0-d_7_/13:0) were purchased from Cayman Chemical. The C13 refers to the carbon chain length for the n-acyl chain for the galactosyl- and dihydroceramide standards. The C15 refers to the carbon chain length for the n-acyl chain for the glucosyl- and lactosylceramides.

#### Lipid extraction

Total lipid extracts from the perilesional cortical tissue samples were prepared using MTBE lipid extraction protocol (22) with slight modifications as described previously (23). Briefly, 400 μl of cold methanol and 10 μl of internal standard mixture (mix EquiSPLASH, 5 fatty acid mixture and 4 ceramides mixtures; the final concentration of the internal standard mixture was 33.3 μg/ml for each lipid) were added to each sample. The sample was incubated at 4°C, 650 rpm shaking for 15 min. Next, 500 μl of cold MTBE was added followed by incubation at 4°C for 1 h with 650 rpm shaking. Cold water (500 μl) was added slowly, and the resulting extract was maintained 4°C, 650 rpm shaking for 15 min. Phase separation was completed by centrifugation at 8,000 *g* for 8 min at 4°C. The upper, organic phase was removed and set aside on ice. The bottom, aqueous phase was re-extracted with 200 μl of MTBE followed by 15 min incubation at 4°C with 650 rpm shaking. Phase separation was completed by centrifugation at 8,000 *g* for 8 min at 4°C. The upper, organic phase was removed and combined with a previous organic extract. The latter was dried under a steady stream of nitrogen at 30°C. The recovered lipids were reconstituted in 100 μl of acetonitrile:isopropanol:water (1:2:1, v/v/v).

Liquid Chromatography Tandem Mass Spectrometry (LC-MS/MS): Total lipid extracts were analyzed by liquid chromatography coupled to targeted tandem mass spectrometry (LC-MS/MS). The LC-MS/MS analyses were performed on an Ultimate 3,000 Ultra High-Performance Liquid Chromatograph coupled to a Thermo TSQ Altis Tandem Quadrupole Mass Spectrometer (Thermo Scientific). LC-MS/MS methodology was adapted from the literature (24) and previous publication (25). The separation was achieved using an ACQUITY Amide BEH column (1.7 μm; 2.1 × 100 mm) column (Waters) maintained at 45°C. Mobile phase compositions for solvents A and B consisted of ACN/H_2_O (95:5, v/v) and (50:50, v/v), respectively, with 10 mM ammonium acetate. The gradient profile had a flow rate of 0.6 ml min^−1^ and ramped from 0.1% to 20% B in 2 min, from 20% to 80% B in 3 min, dropped from 80% to 0.1% B in 0.1 min, and held 0.1% B for 2.9 min. Total chromatographic run time was 8.0 min. The injection volume was 2 μl. The auto-sampler was maintained at 7°C. Electrospray ionization was achieved using either negative or positive mode. Mass spectrometry detection was done using selective reaction monitoring where predetermined precursor to product ion transitions were used. ESI source parameters were set as follows: voltage 3500 V in positive mode and −2500 V in negative mode, sheath gas (Arb) = 60, aux gas (Arb) = 15, sweep gas (Arb) = 1, and ion transfer tube temperature of 380°C. Nitrogen was used as the nebulizer and argon as collision gas (1.5 mTor). The vaporizer temperature was set to 350°C. LC-MS/MS data were acquired using Thermo’s Xcalibur software and data processing was achieved using Xcalibur 4.2 and TraceFinder 5.1. Data were acquired by LC-MS/MS via selection reaction monitoring (SRM); raw data (area counts) was converted to abundance (pmol) per 1-point calibration with class specific internal standard (EquiSplash). The pmol abundance was normalized to tissue amount (pmol/g of tissue). pmol/g was converted to fold change for data visualization. Additional data analysis was done using Prism 6 (GraphPad) and MetaboAnalyst (26).

Lipidomic data has been uploaded to Mendeley data repository with https://doi.org/10.17632/ysymtcyj4s.1. Additional data is available upon request.

### Preparation of crude peroxisomal fraction

Perilesional cortical tissue of saline perfused TBI mice and corresponding cortical tissue region of sham mice were homogenized in buffered ice-cold sucrose solution containing 0.32 M sucrose (Sigma, S9378), 10 mM HEPES (ThermoFisher, 15630080) and protease and phosphatase inhibitors. Homogenates were centrifuged at 1,000 *g* for 10 min at 4°C to spin down nuclei. Supernatants were then centrifuged at 20,000 *g* for 30 min at 4°C to pellet down the crude peroxisomal fractions. Supernatants obtained from this step were further centrifuged at 100,000 g for 1 h at 4°C to isolate the cytosolic fraction (supernatant).

### Western blot analysis

Perilesional area (5 mm) of the ipsilateral cortex of the TBI mice (1 h, 1, 3, 7, and 28 days post-TBI) or the corresponding cortical region of sham mice were dissected and processed as described previously (27). Tissue lysates (15 μg protein) were resolved by gel electrophoresis in 4%–20% SDS-PAGE gels (Bio-Rad, 5671095) and transferred onto PVDF membrane (Millipore, IPVH00010) which was blocked with 5% nonfat milk in tris buffered saline with 0.05% tween 20 (TBST), then probed with primary antibodies in 1% BSA in TBST overnight at 4°C and incubated with HRP-conjugated secondary antibodies (Goat-anti-Rabbit-IgG-HRP-Conjugate, Sigma, 12–348; Goat-anti-Mouse-IgG-HRP-Conjugate, Sigma, 12–349) in blocking solution at room temperature for 1 h. Chemiluminiscence kit (SuperSignal West Pico PLUS Substrate, Thermo Scientific-34095, SuperSigna West Dura Extended Duration Substrate, Thermo Scientific-34077) was used to detect protein bands which were visualized by Chemi-doc system (Bio-Rad) and their intensity was analyzed using Image Lab software (Bio-Rad) and normalized to loading control (β-Actin).

Primary antibodies: AGPS (1:1000; Thermo Scientific, PA5-87935), GNPAT (1:1000; Thermo Scientific, PA5-36447), ABCD3/PMP70 (1:1000, Thermo Scientific, PA1-650), α-Tubulin (1:500; AA4.3-s, developed by Walsh, C. and obtained from Developmental Studies Hybridoma Bank developed under the auspices of the NICHD and maintained by The University of Iowa, Department of Biology, Iowa City, IA 52,242), β-actin/ACTB (1:10,000; Sigma, A1978) and PEX7 (1:1,000, Proteintech, 20614-1-AP) and PEX14 (1:1000, Proteintech, 10594-1-AP).

### Immunohistochemistry

20-μm frozen paraformaldehyde (4% PFA, pH 7.4) fixed sections were obtained from OAG-treated or untreated sham and TBI mouse brains (n = 3/group and 4 sections/mouse) at days 1 or 28 after TBI as previously described (27). Sections were blocked with 5% goat serum (Millipore, S30-100) in 1(X) phosphate-buffered saline (PBS; Quality Biological, INC., 119–069-101) containing 0.025% Triton X-100 (Sigma, T8787), incubated overnight with primary antibodies at 4°C and then with secondary antibodies conjugated with fluorophores in the blocking solution for 2 h at room temperature. Nuclei were stained with DAPI. Immuno-stained sections were imaged using Nikon Eclipse Ti-E/Ni-E microscope. 20X or 60X images were analyzed and quantified by Elements software (V4.12.01, Nikon).

Primary antibodies used include SQSTM1 (1:200; Progen, GP62-C), AGPS (1:250, Thermo Scientific, PA5-87935 and 1:100, Santacruz Biotechnology Inc., sc-374201), NeuN (1:500; Millipore, MAB377), cleaved caspase-3 (1: 200; Cell Signaling Technology; 9661), and PEX14 (1:300, Proteintech, 10594-1-AP), Olig2 (1:100; R&D, AF2418). Secondary antibodies: Alexa Fluor 488 goat anti-rabbit (A11034), Alexa Fluor 633 goat anti-guinea pig (A11075), Alexa Fluor 633 goat anti-mouse (A21052) from Thermo Scientific.

### Real-time PCR

Peri-lesional cortical region (around 5 mm) of TBI mice (1 day and 28 days post-TBI) or the corresponding area of sham mice were dissected and processed to extract RNA using miRNeasy Mini Kit (Qiagen, Cat No. 217004) as described previously (27) and then converted to cDNA using High-Capacity RNA to cDNA kit (Applied Biosystem, Cat. No. 4387406). Quantitative real-time PCR amplification was performed using cDNA TaqMan Universal Master Mix II (Applied Biosystems, Cat. No. 4440040) and 20 × TaqMan® Gene Expression Assay (Applied Biosystems) for the following mouse genes: *Gapdh* (Mm99999915_g1), *Nlrp3* (Mm00840904_m1), *Cybb* (Mm01287743_m1), *Nos2* (Mm00440502_m1), *Tnf* (Mm00443258_m1), *Ifnb1* (Mm00439552_s1), *Il1b* (Mm00434228_m1), *Arg1* (Mm00475988_m1), *Socs3* (Mm00545913_s1), *Chil3* (Mm00657889_mH) and *Il4r* (Mm01275139_m1) (Applied Biosystems). Reactions were performed and the data were analyzed using a 7900HT Fast Real-Time PCR System and corresponding software (Applied Biosystems) respectively. Relative gene expression normalized to *Gapdh* was calculated based on the comparative Ct method (28). For this study, n = 5/group were used for all time points.

### Behavioral methods

A battery behavioral test was performed at time points described in supplemental Fig. S4A. All functional assessment and behavioral tests were carried out and scored blinded as described previously.

#### Beam walk test

Motor co-ordination was assessed in sham and OAG-fed or -unfed TBI mice on Days 1, 3, 7, 14, 21, and 28 post injury using beam walk test as described previously (20, 21).

#### Morris Water Maze (MWM) test

Hippocampal-dependent spatial learning and memory in sham and OAG-fed or -unfed TBI mice were assessed by MWM test at PID 21–25 as described, previously (21, 29). Data were acquired and analyzed using Any-Maze software.

### Lesion volume

Cortical lesion volume in OAG-fed or -unfed injured mouse brains at PID 28 was determined using Cavalieri method sing Stereo-investigator software, version 2020.2.3 (MBF Biosciences) as per the methods described previously.

## Statistics

All quantitative data are presented as mean ± standard error of the mean (SEM) and were analyzed using GraphPad Prism program, 10.4.0 for Windows (GraphPad Software) for statistical significance (*P* value ≤ 0.05). The number of mice used was determined by Power analysis (power of 0.8; alpha value 0.05). Mouse distribution into different groups and time points were performed randomly. A two-tailed student's *t* test with equal variance was used for comparing between two groups and one- or two-way ANOVA followed by appropriate post-hoc tests were used for comparison of more than two groups. A two-way ANOVA with repeated measure was performed for beam walk analysis.

## Results

### Ether-GPs decrease in the mouse cortex after TBI

**Fig. 1:**
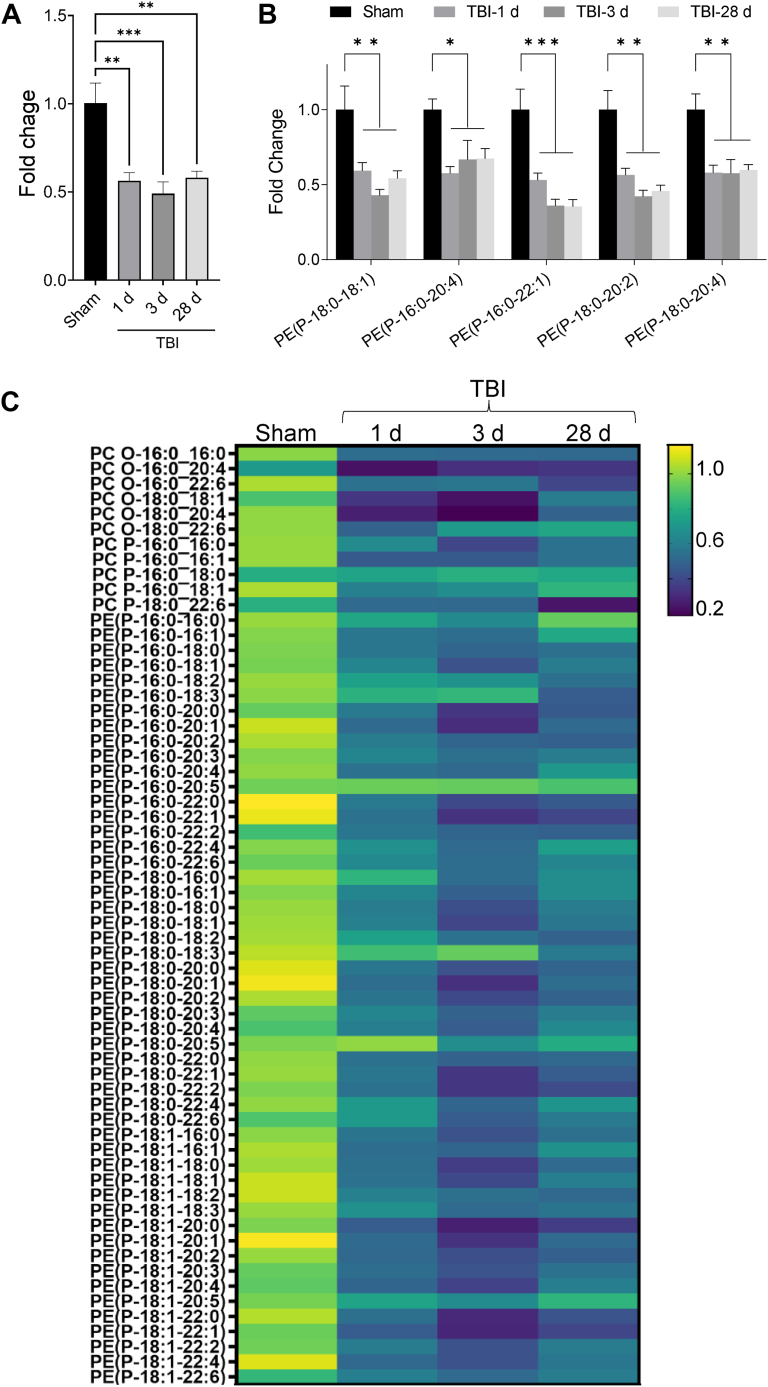
Ether-GPs abundance decreased after TBI. A: Bar diagram representing time-dependent changes in the ether-GPs abundance in the mouse cortices after TBI. Data presented as mean ± SEM. n = 5; ∗∗*P* < 0.01 and ∗*P* < 0.05 with respect to sham determined by One-way ANOVA. B: Bar diagram showing the time-dependent decrease in different plasmalogens' abundance in the injured mouse cortices after TBI. Data presented as mean ± SEM. n = 5; ∗∗∗*P* < 0.001, ∗∗*P* < 0.01 and ∗*P* < 0.05 with respect to sham determined by One-way ANOVA. C: Heatmap displaying changes in the abundance of ether-phosphatidylcholine (ePC) and ether-phophatidylethanolamine (ePE) in the perilesional area of the mouse cortices after TBI. Data presented as fold change.

To understand how ether-GPs are affected by TBI we determined the levels of ether-GPs in the ipsilateral cortical tissue of mice following CCI-induced moderate TBI using liquid chromatography tandem mass spectrometry (LC-MS/MS). Our data showed that overall cortical ether-GPs abundance decreased in the injured as compared to sham mice at days 1 and 3 and remained low till day 28 after TBI (Fig. 1A). We observed that persistent significant decline in many vinyl-ether-GPs (plasmalogens) abundance in the mouse cortices after TBI (Fig. 1B, C). As expected, we also detected a decrease in total glycerophospholipids after TBI (supplemental Fig. S2). This suggests a broad-based long-term decline in ether-GPs level in the cortical tissue following brain injury.

### TBI causes decrease and mislocalization of peroxisomal ether-GPs synthesizing enzymes

**Fig. 2:**
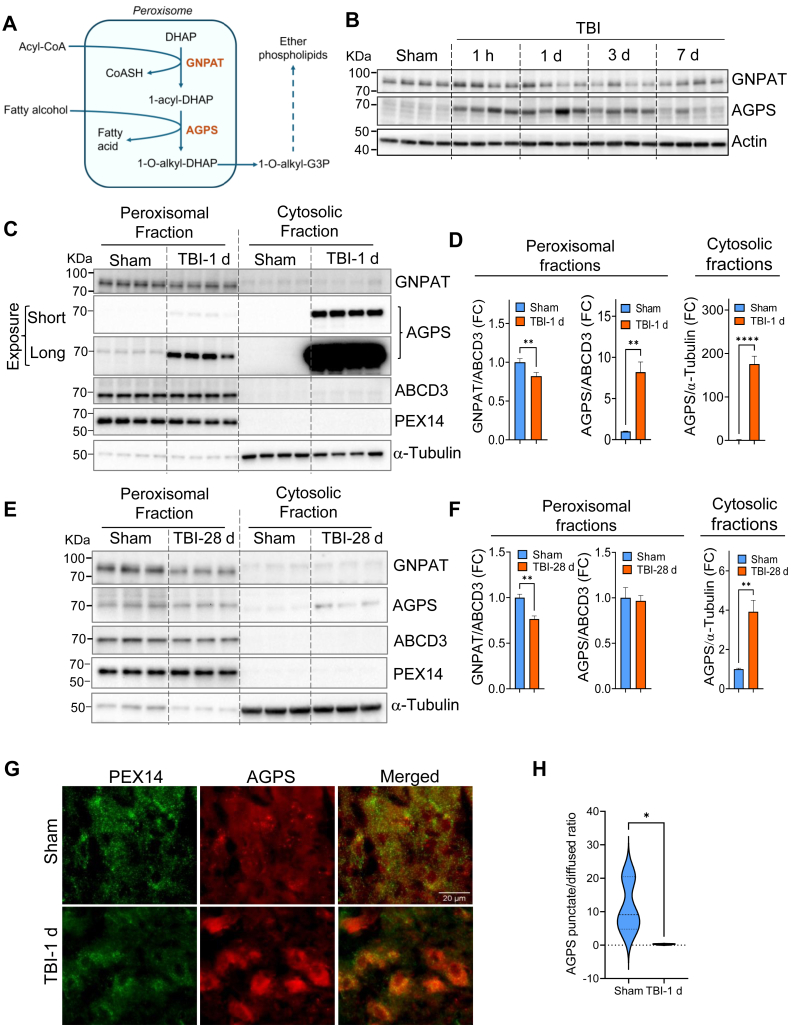
Peroxisomal ether-GPs synthesis was disrupted after TBI. A: schematic diagram showing peroxisomal ether-GPs synthesizing steps. It is initiated by the acylation of dihydroxyacetone phosphate (DHAP) by GNPAT that generates 1-acyl-DHAP which is then converted to 1-O-alkyl-DHAP by AGPS. 1-O-alkyl-DHAP is then converted to 1-O-alkylglycerol phosphate which is transported to the endoplasmic reticulum for the generation of fully formed ether-GPs. B: Western blots of peroxisomal enzymes GNPAT and AGPS in the cortical tissue lysates of sham and TBI mice. Data presented as mean ± SEM. n = 4; ∗∗*P* < 0.01 and ∗*P* < 0.05 with respect to sham determined by One-way ANOVA. C: Western blots and (D) corresponding quantification of peroxisomal enzymes GNPAT and AGPS in the peroxisomal and cytosolic fractions prepared from the sham and TBI (PID 1) mouse cortices. ABCD3 (PMP70) and PEX14 are markers of peroxisomal membrane and α-tubulin is of cytosolic fraction. Data presented as mean ± SEM. n = 4. ∗∗*P* < 0.01 and ∗*P* < 0.05 with respect to sham determined by One-way ANOVA. E: Western blots and corresponding (F) quantification of GNPAT and AGPS in the peroxisomal (ABCD3+) and cytosolic (α-tubulin+) fractions of sham and TBI mouse cortices (PID 28). Data = Mean ± SEM; n = 5. ∗∗*P* < 0.01, Students' t test. G: 60X images of AGPS and peroxisomal membrane marker PEX14. H: Quantification of punctate versus diffused AGPS ratio in sham (blue) and injured (1 day after TBI) (red) brain sections. Data presented as mean ± SEM. n = 3; ∗*P* < 0.05; Students' t test.

Ether-GP synthesis is initiated within peroxisomes. Their ether-bond is generated by the concerted action of peroxisomal enzymes—glyceronephosphate-N-acyltransferase (GNPAT) and alkylglycerone phosphate synthase (AGPS) (Fig. 2A). GNPAT catalyzes the acylation of dihydroxyacetone phosphate. The acyl group is replaced by an alkyl group generating the ether bond in the following step which is catalyzed by AGPS (1, 4, 5, 6, 7). We hypothesized that lower abundance of ether-GPs after injury could be due to a defect in peroxisomal ether-GPs synthesis and examined expression of GNAPT and AGPS in the mouse cortices after TBI. We found that the mRNA levels of *Gnpat* and *Agps* remained mostly unaltered as compared to sham mice (supplemental Fig. S3), with only a small increase in *Gnpat* mRNA at post injury day 7 (supplemental Fig. S3). Our Western blot data showed a slight decline in GNPAT protein level in the mouse cortices after TBI (Fig. 2B and supplemental Fig. S4A). On the contrary, we observed a rapid increase in AGPS protein level in the acute TBI phase starting from 1 h after injury (Fig. 2B and supplemental Fig. S4B). No significant changes in GNPAT and AGPS levels were detected on day 28 after TBI (supplemental Fig. S4C, D). These data suggest that these enzymes are differentially regulated over time at post-transcriptional level in the mouse cortices following TBI.

Next, we determined subcellular distribution of the peroxisomal enzymes in the cortices of sham and TBI mice. We fractionated cortical tissues from sham and injured mice at 1 day and 28 days after TBI into crude peroxisomal and cytosolic fractions and determined the levels of GNPAT and AGPS in those fractions by Western blots. Our data showed a significant decline in GNPAT level in the peroxisomal fractions of TBI mice at both post injury days (PIDs) 1 (Fig. 2C, D) and 28 (Fig. 2E, F) as compared to sham. GNPAT level was almost undetectable in the cytosolic fractions. Consistent with its overall accumulation after injury, we detected markedly elevated levels of AGPS in both peroxisomal and cytosolic fractions prepared from the injured mouse cortices at PID 1 (Fig. 2C, D). However, AGPS accumulation in the cytosol was much more prominent than that detected in the peroxisomal fraction (175-fold vs. 8-fold, respectively). Additionally, while levels of peroxisomal AGPS returned to the sham level by 28 PID, they remained significantly elevated in the cytosol (Fig. 2E, F). These data indicate that while the protein levels of AGPS are increased after TBI, it is mis-localized away from peroxisomes. This was further confirmed by immunofluorescence (IF) staining experiment. We observed that while AGPS predominantly appeared as punctate structures which mostly colocalize with peroxisomal membrane marker—PEX14, it was diffuse in many cells in the cortices of TBI mice indicating its cytosolic accumulation (Fig. 2G, H). Since AGPS is transported to peroxisomes by peroxisomal biogenesis factor—PEX7 (30, 31), we determined its level in sham and TBI mouse cortices to detect whether AGPS cytosolic accumulation after TBI is caused due to the defect in its PEX7-mediated transport. Our data showed no significant change in PEX7 level in TBI mouse cortices suggesting that AGPS accumulation might be caused by other mechanisms (supplemental Fig. S5). Together, cytosolic mis-localization of AGPS and reduced peroxisomal level of GNPAT suggest that peroxisomal ether-GPs synthesizing enzymes are dysregulated causing reduction in ether-GPs abundance after TBI.

### Peroxisomal dysfunction is associated with autophagic defect and neuronal apoptosis

**Fig. 3:**
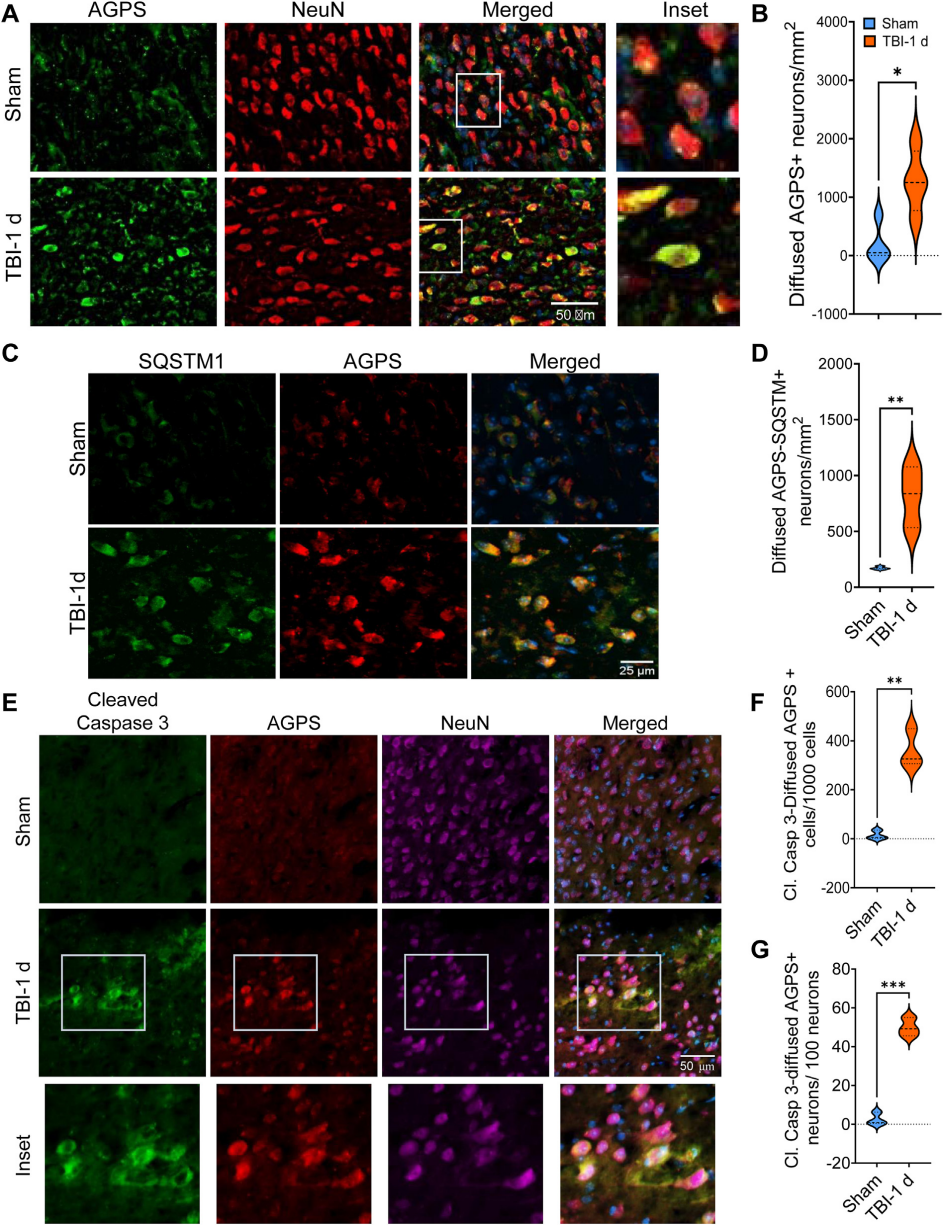
AGPS accumulation was associated with autophagic disruption and neuronal apoptosis after TBI. A: 20X images of sham and TBI mouse cortical brain sections stained with AGPS and neuronal marker NeuN. B: Quantification of diffused AGPS positive neurons in the sham (blue) and injured (1 day after TBI) (red) brain sections. Data presented as mean ± SEM. n = 3; ∗*P* < 0.05; Students' t test. C: 20X images of sham and TBI mouse cortical brain sections stained with AGPS and autophagic cargo/adapter protein – p62/SQSTM1. D: Quantification of diffused AGPS and p62/SQSTM1 positive cells in the sham (blue) and injured (1 day after TBI) (red) brain sections. Data presented as mean ± SEM. n = 3; ∗∗*P* < 0.01; Students’ t test. E: 20X images of sham and TBI mouse cortical brain sections stained with Agps, apoptotic cell death marker – cleaved caspase 3 and neuronal marker – NeuN. F, G: Quantification of diffused Agps and cleaved caspase 3 positive cells (F) and neurons (G) in the sham (blue) and injured (1 day after TBI) (red) brain sections. Data presented as mean ± SEM. n = 3; ∗∗∗*P* < 0.001, ∗∗*P* < 0.01; Students' t test.

We previously demonstrated that autophagy, a cellular catabolic pathway that degrades damaged dysfunctional proteins and organelles including peroxisomes, was disrupted in the mouse cortices that contributes to neuronal death after TBI (20, 27). In the current study, our data showed that AGPS predominantly accumulated within neurons in the injured mouse brains (Fig. 3A, B). We also detected its moderate accumulation in oligodendrocytes (supplemental Fig. S6A, B). However, the number of oligodendrocytes with diffused AGPS was significantly lower as compared to other cells in which AGPS accumulated after TBI (supplemental Fig. S6A, C). This suggests that AGPS accumulates predominantly within neurons after TBI. Thus, we hypothesized that increased accumulation of AGPS might be due to impaired autophagic clearance after brain injury. Autophagic disruption after TBI causes accumulation of autophagosome and its cargo adaptor protein p62/SQSTM1 (20, 27). To detect if the increased cytosolic levels of AGPS are associated with autophagic disruption, we performed IF staining for p62/SQSTM1 and AGPS in the sham and TBI mouse brain sections. Our data showed that the cells in which AGPS accumulated also had elevated levels of p62/SQSTM1 (Fig. 3C, D). This suggests that AGPS accumulation after TBI might be due at least in part to the impairment of autophagy.

TBI causes acute and progressive neuronal death. We determined whether AGPS accumulation may be associated with neuronal apoptosis after brain injury. We stained the sham and injured brain sections for AGPS, and the apoptotic cell death marker—cleaved Caspase 3. We observed that in injured cortices, cells with higher level of diffuse cytosolic signal for AGPS were frequently positive for cleaved Caspase 3 (Fig. 3E, F). We also detected cleaved caspase 3 signals in many neurons with diffused AGPS (Fig. 3E–G). This suggests that AGPS accumulation is associated with neuronal apoptosis after TBI.

### Ether-GPs precursor supplementation partially restored ether-GPs levels after TBI

**Fig. 4:**
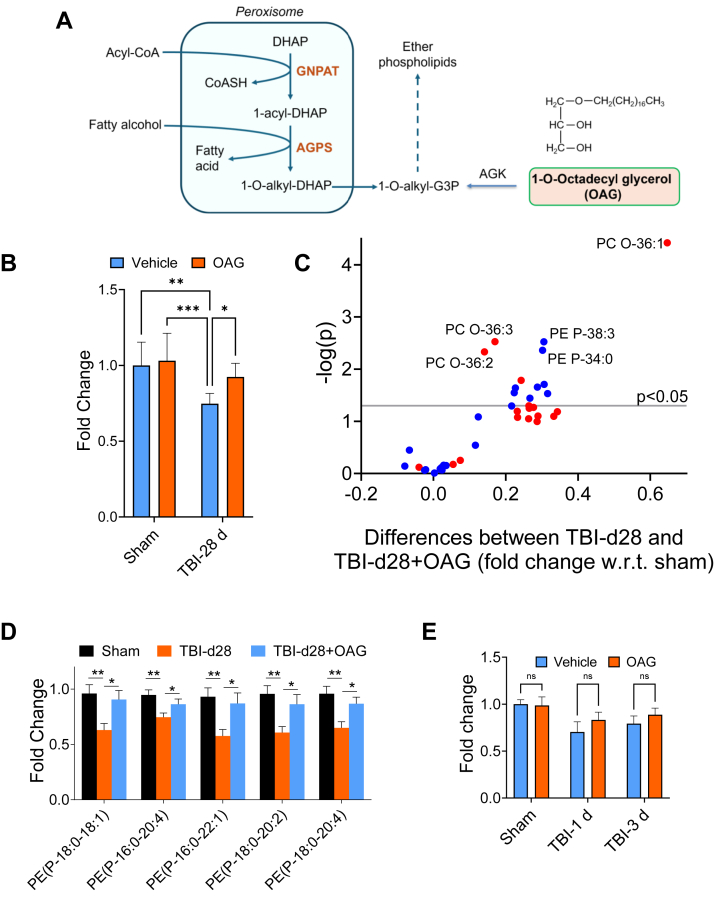
OAG treatment restored ether-GPs abundance in the injured cortices. A: schematic diagram showing how 1-O-octadecylglycerol (OAG) restores ether-GPs abundance in the injured brains. Alkylglycerol kinase (AGK) can phosphorylate 1-O-octadecylglycerol (OAG) to 1-O-octadecylglycerol phosphate, which can bypass peroxisomal ether-GPs synthesizing steps and can restore ether-GPs abundance in the injured brain. B: Bar diagram showing ether-GPs abundance in the cortices of sham and injured mice treated with or without OAG. Data presented as mean ± SEM. n = 6; ∗∗∗*P* < 0.001, ∗∗*P* < 0.01 and ∗*P* < 0.05 determined by Two-way ANOVA. C: Volcano plot showing the differences in fold change in ether-GPs abundance in the cortices of TBI mice treated with OAG and those of untreated TBI mice. Red dots represent alkyl and blue dots represent alkenyl ether-GPs. The abundance of ether-GPs species above the gray line (*P* < 0.05) was significantly altered in the injured mouse cortices by OAG treatment. The location of selected lipid species of interest is indicated. The x-axis represents the differences in fold change (FC) in ether-GPs abundance between OAG-treated and untreated TBI mice, and the y-axis is – log10(p) (p = *P*-value based on *t* test). n = 6 mice/group. D: Bar diagram showing restoration of different plasmalogens' abundance in the injured cortices of mice treated with OAG. Data presented as mean ± SEM. n = 5; ∗∗*P* < 0.01 and ∗*P* < 0.05; One-way ANOVA. E: Bar diagram showing ether-GPs abundance in the cortices of sham and injured mice treated with or without OAG. Data presented as mean ± SEM. n = 6. No significant changes in ether-GPs abundance between OAG-treated and untreated mice were detected at post-injury days 1 and 3.

Ether-GPs synthesis is carried out by concerted functions of peroxisomes and endoplasmic reticulum. It is initiated within peroxisomes that generate ether-GPs precursor 1-O-alkyl glycerol which is transferred and converted to ether-GPs in the ER (1, 4, 5, 6, 7). Since our data indicated that the decrease in ether-GPs abundance in the injured mouse cortices is at least in part caused by the impairment in peroxisomal ether-GPs synthesis, we hypothesized that ether-GPs levels could be restored by bypassing peroxisomal ether-GPs synthesizing steps (Fig. 4A). This can be achieved by treating mice with ether-GPs precursors that are generated by peroxisomes. We used 1-O-octadecyl glycerol (OAG), oral administration of which has been shown to increase ether-GPs levels in different tissues of a mouse model deficient in peroxisomal ether-GPs synthesis (32). Since blood–brain barrier is disrupted after TBI, we expected that OAG could easily reach the injured brain area and restore ether-GPs levels therein. To ensure that ether-GPs levels remain elevated and can provide neuroprotection at the very early stage of injury, mice were administered OAG supplemented diet starting one week prior to CCI-induced TBI (supplemental Fig. S7A). No major change in their body weight was detected as compared to mice fed with standard diet either with or without injury (supplemental Fig. S7B).

We determined the levels of ether-GPs in the cortices of the treated mice using LC-MS/MS. Our data showed a significant cumulative increase in ether-GPs, including both ether-PC and -PE levels, in the injured cortices of mice fed OAG-supplemented diet 28 days after TBI (Fig. 4B). At that time point we also detected significant upregulation in levels of individual ether-GPs in the injured cortices of OAG-fed mice as compared to untreated TBI mice (Fig. 4C, D). However, no significant changes were detected in any ether-GPs in OAG-fed mice at early time points (Days 1 and 3) after TBI (Fig. 4E). This could be due to highly elevated levels of oxidative stress in the cortical tissues of TBI mice at the early stage of injury leading to oxidation and degradation of both endogenous and exogenous vinyl ether-GPs.

### Ether-GPs precursor treatment improved recovery after TBI

**Fig. 5:**
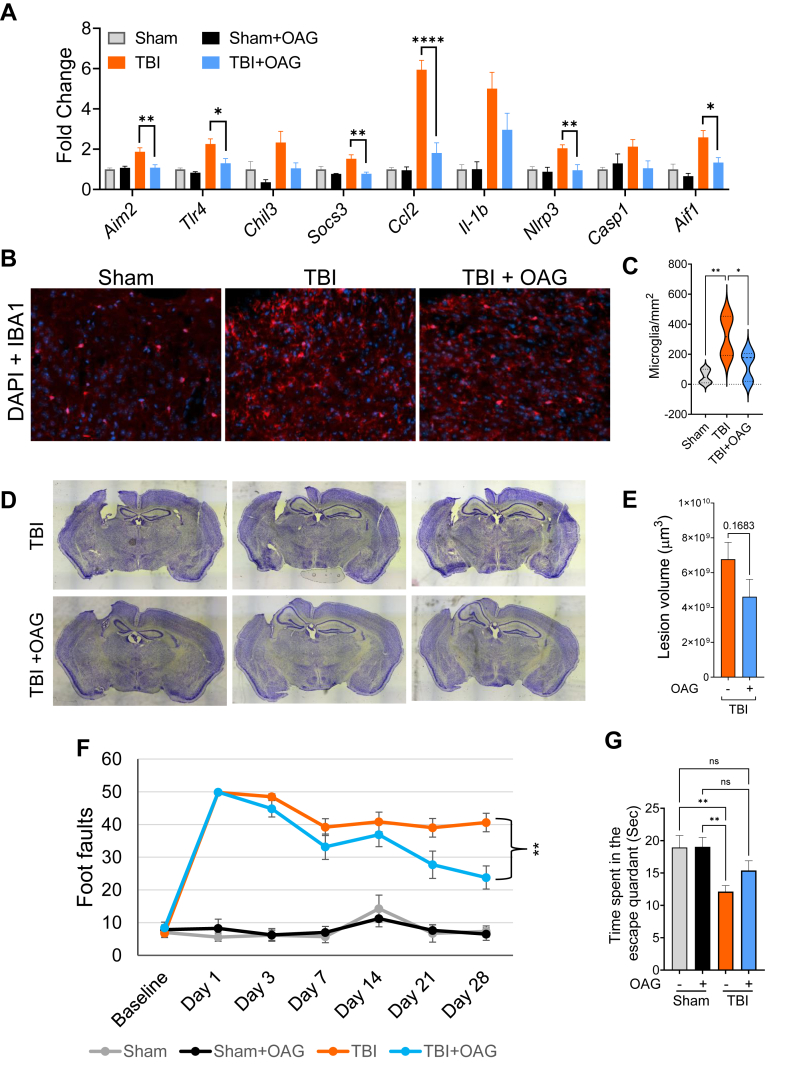
OAG treatment attenuated neuroinflammation, cortical tissue loss and functional deficits in TBI mice. A: OAG treatment reduced expression of different inflammatory markers in the injured cortices of TBI mice on post injury day 28. Data presented as mean ± SEM. n = 6; ∗∗∗∗*P* < 0.0001; Two-way ANOVA. B: 20 X images of sham and OAG-treated and -untreated TBI mouse brain sections stained with microglial marker IBA1. C: Quantification of IBA1 positive microglial cells in sham (gray) and OAG-treated (red) and -untreated (blue) TBI mouse cortical sections. D: Representative images of lesion volume at 28 days post injury in OAG-untreated and -treated TBI mouse cortices. E: Stereological (Cavalieri method) quantification of lesion volume in OAG-untreated and -treated TBI mouse cortices. Data are presented as mean ± SEM. n = 5/group. F: Sensory-motor function was assessed in sham and TBI mice treated or untreated with OAG by beam walk test. The number of hindlimb foot faults per 50 steps was counted on days 0, 1, 3, 7, 14, 21 and 28 after TBI. Significant injury and treatment effects were detected across all time points except day 0 (baseline, prior to injury). (Two-way repeated-measures ANOVA with Tukey's multiple comparison test). Both OAG-treated and untreated TBI groups showed significant difference in motor function (foot faults) as compared to the sham groups at all time points except day 0 (^#^*P* < 0.01). Significant improvement in motor function was detected in OAG-fed TBI group as compared to the untreated TBI mice on day 28 (∗∗*P* < 0.01). Data are presented as mean ± SEM, n = 7 Sham, 7 Sham + OAG, 10 TBI and 10 TBI + OAG. G: Cognitive function in sham and TBI mice treated with or without OAG was assessed using Morris water maze (MWM). The bar diagram represents time spent by sham and TBI OAG-untreated and treated mice in the escape quadrant on the probe trial day following a 4-days of training. Both OAG-treated and untreated mice spent less time than the sham mice. OAG-treated TBI mice spent slightly more time in the escape quadrant than the untreated TBI mice. Data = Mean ± SEM; ∗∗*P* < 0.01, Two-way ANOVA. n = 16 Sham, 15 Sham + OAG, 18 TBI and 16 TBI + OAG.

Next, we assessed whether the OAG-induced restoration of ether-GPs had neuroprotective effects in TBI. TBI causes progressive cell death and neuroinflammation in the brain. First, we determined the levels of pro-inflammatory cytokines in cortices of TBI mice with and without OAG supplementation. We observed a small decline in inflammatory markers, including *Cgas*, *Nlrp3*, *Chil3*, *Tlr4*, and *Il4ra* in OAG-treated mice at the early phase of injury (3 days after TBI) (supplemental Fig. S8). However, at the later time point (PID 28), many of the tested inflammatory markers decreased significantly in the OAG supplemented as compared to normal diet-fed TBI group (Fig. 5A). This included significant downregulation in microglial marker *Aif1* (encoding IBA1) expression, suggesting that OAG attenuated microglial proliferation in the injured cortices. This was confirmed by IF staining. We counted significantly lower numbers of IBA1-positive microglial cells in the OAG-treated TBI mouse brains than in untreated injured mice (Fig. 5B, C). These data indicate that restoration of ether-GPs by OAG treatment attenuates neuroinflammation after TBI. We also determined that OAG treatment restricted overall cortical tissue loss in TBI mice, as indicated by reduction in lesion volume in TBI mice treated with OAG as compared to control diet (Fig. 5D, E).

TBI-induced neuronal loss and neuroinflammation severely affects motor and cognitive functions (21). We assessed if restoration of ether-GPs by OAG-supplementation could attenuate TBI-caused functional deficits. We assessed motor function in TBI mice using beam walk test. Our data showed a gradual improvement in beam walk performance in OAG-treated TBI mice as compared to the untreated injured mice (Fig. 5F). We detected a significant improvement in motor function at PIDs 21 and 28 in OAG-treated mice. To determine the effects of ether-GPs-restoration on their cognitive functions, we tested spatial memory using Morris Water Maze test (Fig. 5G). We observed a significant decline in memory in TBI mice on standard diet. However, OAG-supplemented injured mice did not show significant decline in memory function as compared to sham. They demonstrated a modest improvement in their memory function as compared to untreated TBI mice. Taken together these data demonstrate that ether-GPs-restoration by OAG supplementation improves functional recovery in TBI mice.

## Discussion

The pathophysiology of TBI is extremely complex. It causes rapid alteration in multiple metabolic functions that persist chronically causing progressive neurodegeneration and neuroinflammation and potentially lifelong disability (17, 18, 19, 20). Understanding these changes is extremely important for the development of effective treatment strategies. In this study, we demonstrate for the first time that ether-GPs homeostasis is disrupted in the brain following TBI. We found that ether-GPs abundance declined rapidly and remained low for almost a month after TBI, suggesting that ether-GPs dysregulation persists chronically in the injured brain. This is supported by a previous report describing reduced ether-GPs in the mouse cortex and cerebellum after TBI (33). We observed that the abundance of both alkyl and alkenyl (plasmalogens) ether-GPs decreased persistently in the injured brain. Plasmalogens are major components of neuronal membrane and play an important role in synaptic vesicle cycling. All ether-GPs provide structural rigidity to the neuronal membranes, constitute lipid rafts and maintain cellular signaling pathways (1, 7, 8, 9). Reduced levels of ether-GPs adversely affect neuronal function. We expect that TBI-induced decline in ether-GPs level significantly contributes to the chronic functional impairment after brain injury. We found that partial restoration of brain ether-GPs level in TBI mice by the supplementation with ether-GPs precursor OAG, improved their functional recovery. The improvement was particularly notable in motor function, suggesting that motor neurons may be more susceptible to changes in ether-GPs levels. Since ether-GPs are major components of myelin, this could be due to the de-/hypo-myelination of motor neurons because of the reduced ether-GPs levels after TBI.

We found that the decrease in ether-GPs in the injured brain was at least in part caused by impairment of peroxisomal steps of ether-GPs synthesis. Peroxisomes are necessary for the generation of ether-bond in ether-GPs which are catalyzed by GNPAT and AGPS. We found that AGPS accumulated in the cytosol after TBI. This could be due to peroxisomal membrane damage leading to leakage of peroxisomal proteins into the cytosol, or by impairment in peroxisomal protein import. Based on our data, its PEX7-mediated transport is probably not affected after TBI. However, this requires further detailed investigation as AGPS transport could be affected at other steps. We will explore this in our future study. Our data showed that cytosolic accumulation of AGPS was associated with autophagic disruption in the injured cortices. These suggest that AGPS turnover may be regulated by autophagy. Unlike AGPS, levels of another peroxisomal enzyme, GNPAT, decreased rather than increased in the peroxisomal fractions from the injured mice. This suggests that while AGPS levels are regulated by autophagy, GNPAT turnover may be regulated by different mechanisms. Autophagy also plays an important role in clearing nonfunctional damaged peroxisomes by a process called pexophagy (34, 35, 36). We previously demonstrated that autophagy is impaired after TBI (20, 27) suggesting that pexophagy might be disrupted in the injured brain causing accumulation of damaged nonfunctional peroxisomes. Since peroxisomes can proliferate by growth and division of the existing peroxisomes (37, 38), accumulation of damaged peroxisomes can affect the generation of new functionally active peroxisomes. This could also contribute to ether-GPs’ decline after TBI. However, other possible mechanisms might also be involved. TBI is associated with acute oxidative stress which may contribute to ether-GPs decline. Ferroptosis, an iron dependent lipid oxidation mediated cell death pathway has been shown to be activated after TBI and thus may contribute to ether-GPs decline in the injured brain (39). However, ferroptosis has been linked to the increase in peroxisomal ether-GPs synthesis in several in vitro studies (40). This is in contrary to our findings that showed impairment in peroxisomal ether-GPs synthesis in the injured cortices suggesting that ferroptosis may not be the primary cause for ether-GPs decrease in the injured brain. Additionally, phospholipases which are activated after TBI (20, 41) might also be involved in degrading ether-GPs and lowering their level in the injured brain. We previously reported cPLA2 activation in the mouse brain in the early acute stage of injury (1h–3 days post injury). In the current study, we observed that ether-GP declined persistently and remained low in the mouse brain at least up to day 28 post injury. We observed AGPS mislocalization also persisted till day 28 post TBI. This might be causing persistent impairment in peroxisomal ether-GP synthesis and thus contributing at least in part to long-term ether-GP decline after TBI.

Ether-GPs precursors have been shown to modulate endogenous ether-GPs abundance in animal models (32, 42, 43, 44). Alkylglycerols can bypass the peroxisomal steps of ether-GPs synthesis and increase ether-GPs abundance in different tissues. However, many studies reported a very small or no increase of brain ether-GPs level following alkylglycerol treatment in mice (32, 44). This could be due to the poor bioavailability of alkylglycerol to the brain because of the blood–brain barrier. In this study, we used OAG to restore ether-GPs decline following TBI. While we did not observe a significant increase in ether-GP levels in the cortices of OAG-treated sham mice, we detected partial restoration of ether-GPs abundance in TBI mice following prolonged treatment with OAG. It is possible that TBI-caused disruption of blood–brain barrier might have facilitated entry of OAG into the injured brain. Another possibility could be OAG-mediated modulation of ether-GPs abundance in blood monocytes which infiltrate the injured brain. Both neutrophil and macrophages are highly enriched with ether-GPs, particularly plasmalogens (45, 46). Changes in both alkyl- and alkenyl (plasmalogens)-ether-GPs may induce membrane remodeling that favors inflammatory response. OAG-mediated increase in ether-GPs abundance in blood monocytes could attenuate these inflammatory responses as well as contribute to cortical ether-GPs restoration after TBI.

Interestingly, while we expected P-18:0 ether-GP structure to be most impacted following OAG-treatment, we also detected an elevated level of vinyl ether P-16:0 in OAG-fed TBI mouse cortices. This indicates that ether-GP synthesis was broadly impacted by OAG treatment in TBI mice and suggests that OAG-supplementation is effective for restoring vinyl ether lipids in general and not just impactful for specific structures. Additionally, it could also be partly due to attenuation of neuroinflammation in TBI mice following OAG-treatment. TBI is associated with local and systemic inflammation that triggers activation of brain resident microglia and peripheral macrophages (29, 47). Inflammation is known to alter cellular glycerophospholipid abundance and generate inflammatory lipid metabolites (48). Our data showed that OAG treatment attenuated neuroinflammation in TBI mouse brains. Restoration of broad ether-GP (of varied chain length) abundance in the injured mouse brain following OAG-treatment might also be due to the attenuation of inflammation.

Decreased ether-GPs abundance has been linked to microglial activation. LPS and IL-1β mediated microglial activation is associated with the reduced ether-GPs level (49, 50), while reduction in plasmalogens level by the knockdown of GNPAT activates microglia. Conversely, plasmalogen treatment has been shown to reduce microglial activation in aged mice (51) and AD-mouse models (50, 52). We found that the restoration of ether-GPs by OAG supplementation can attenuate microglial numbers and inflammatory cytokine expression in the injured brain. This suggests that decrease in ether-GPs after TBI contributes to the inflammatory responses of microglia and macrophages. Since ether-GPs impart rigidity to the membrane, reduced ether-GPs levels may be involved in promoting phagocytosis in the injured brain by increasing membrane fluidity. Additionally, decreased membrane ether-GPs level may also promote inflammatory signaling in the injured brain.

Decrease in ether-GPs abundance has been reported in neurodegenerative diseases, suggesting that ether-GPs decline is a common phenotype in neurological disorders. In AD, decrease in ether-GPs has been attributed to the reduced expression of AGPS (1, 10). Here we observed AGPS dysregulation in the mouse cortex after TBI. This represents mechanistic similarity of peroxisomal dysfunction in ether-GPs synthesis between TBI and AD. This suggests that impairment in peroxisomal ether-GPs synthesis might be a common mechanism for ether-GPs decrease across different neurodegenerative diseases. This also suggests that improving peroxisomal function or restoring ether-GPs abundance might be beneficial not only in TBI but also in other neurodegenerative diseases.

## Data availability

Lipidomic data has been uploaded to Mendeley data repository with https://doi.org/10.17632/ysymtcyj4s.1. All other data are provided in the manuscript.

## Supplemental data

This article contains supplemental data.

## Conflict of interest

The authors declare that they have no conflicts of interest with the contents of this article.
