# Supplementary material for: Peroxisomal ether-glycerophospholipid synthesis is dysregulated after TBI
Source: J Lipid Res. 2025 May 7;66(6):100821. doi: 10.1016/j.jlr.2025.100821 (PMC12255425; doi:10.1016/j.jlr.2025.100821)
Supplement: Supplemental Figures [file mmc1.pdf]

Figure –S1

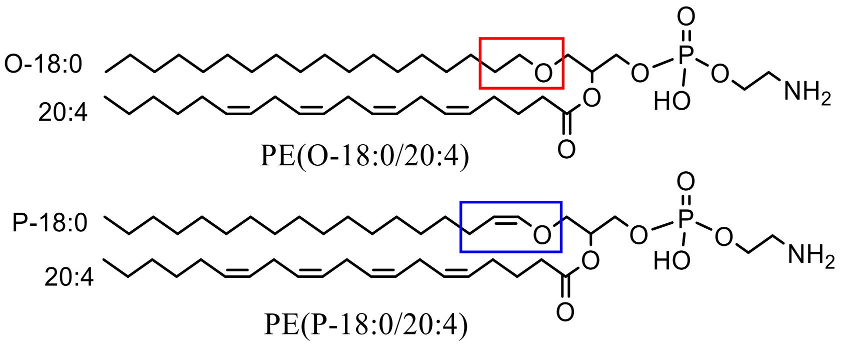

Supplemental figure 1. Representative structures of alkyl [PE(O-18:0/20:4)] and alkenyl -ether-GP [PE(P-18:0/20:4)].

Figure –S2

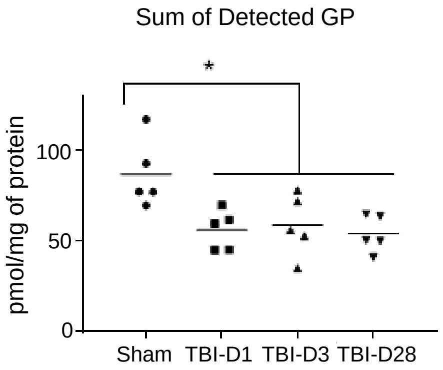

Supplemental figure 2. Changes in total glycerophospholipid (GP) abundance in sham and TBI mouse cortices. *p<0.05, One-way ANOVA. n=6.

Figure –S3

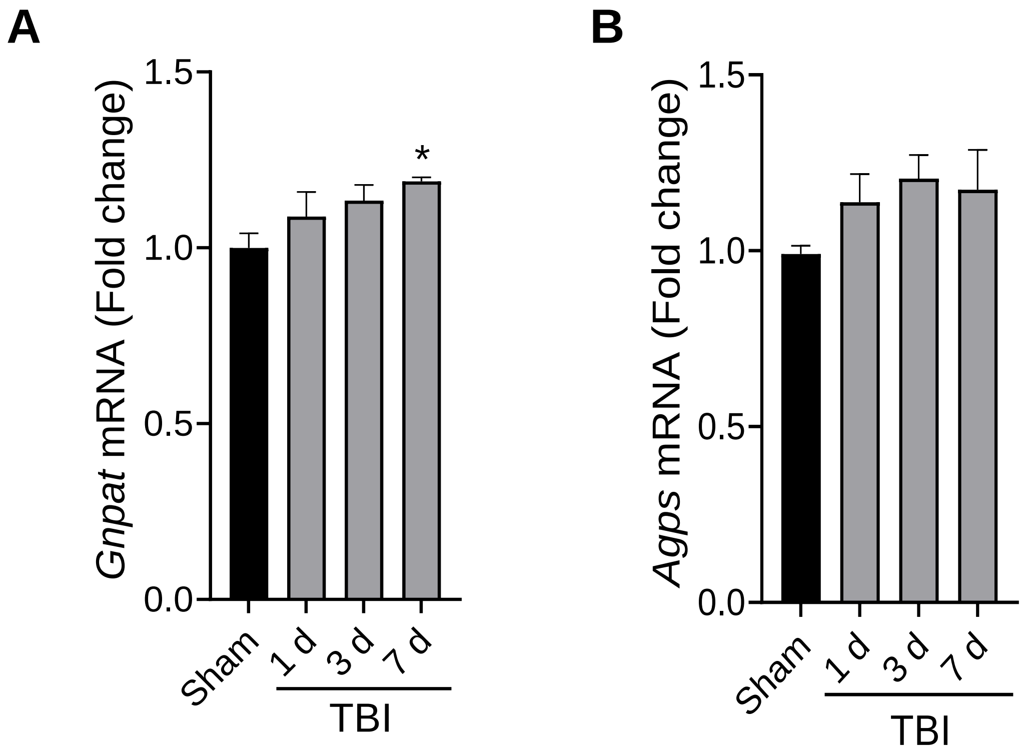

Supplemental figure 3. *Gnpat* and *Agps* expression after TBI. Relative mRNA levels of **(A)** *Gnpat* and **(B)** *Agps* normalized to *Gapdh* mRNA in the mouse cortices after TBI. Data=Mean ± SEM; n=6; *P<0.05, One-way ANOVA.

Figure –S4

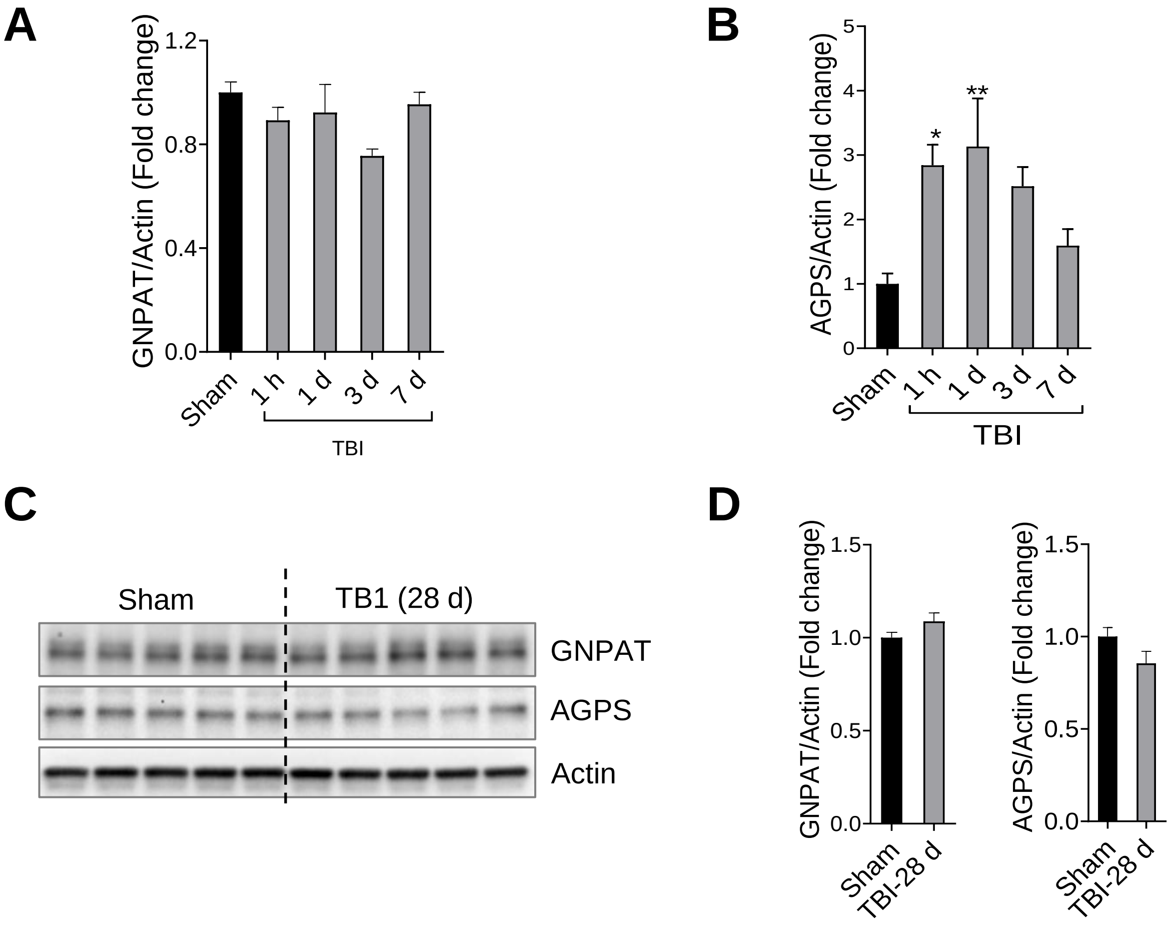

Supplemental figure 4. GNPAT and AGPS protein levels in the injured mouse cortices. Densitometric quantification of the Western blots of **(A)** GNPAT and **(B)** AGPS in the sham and TBI mouse cortices. **(C)** Western blots and corresponding **(D)** quantification of GNPAT and AGPS in the sham and TBI mouse cortical tissue lysates (PID 28). Data=Mean ± SEM; n=5. Data presented as mean ± SEM. n=4; **P<0.01 and *P<0.05 with respect to sham determined by One-way ANOVA.

Figure –S5

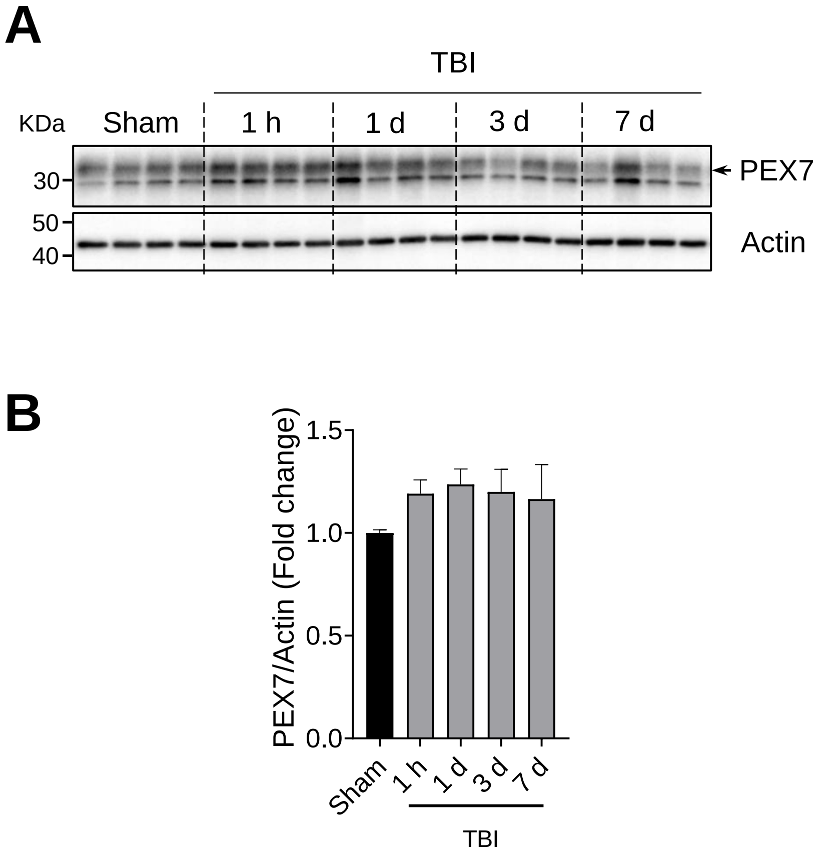

Supplemental figure 5. PEX7 level in the injured cortical tissue. (A) Western blot and **(B)** corresponding quantification of PEX7 in sham and TBI mouse cortices. Data presented as mean ± SEM. n=4.

Figure –S6

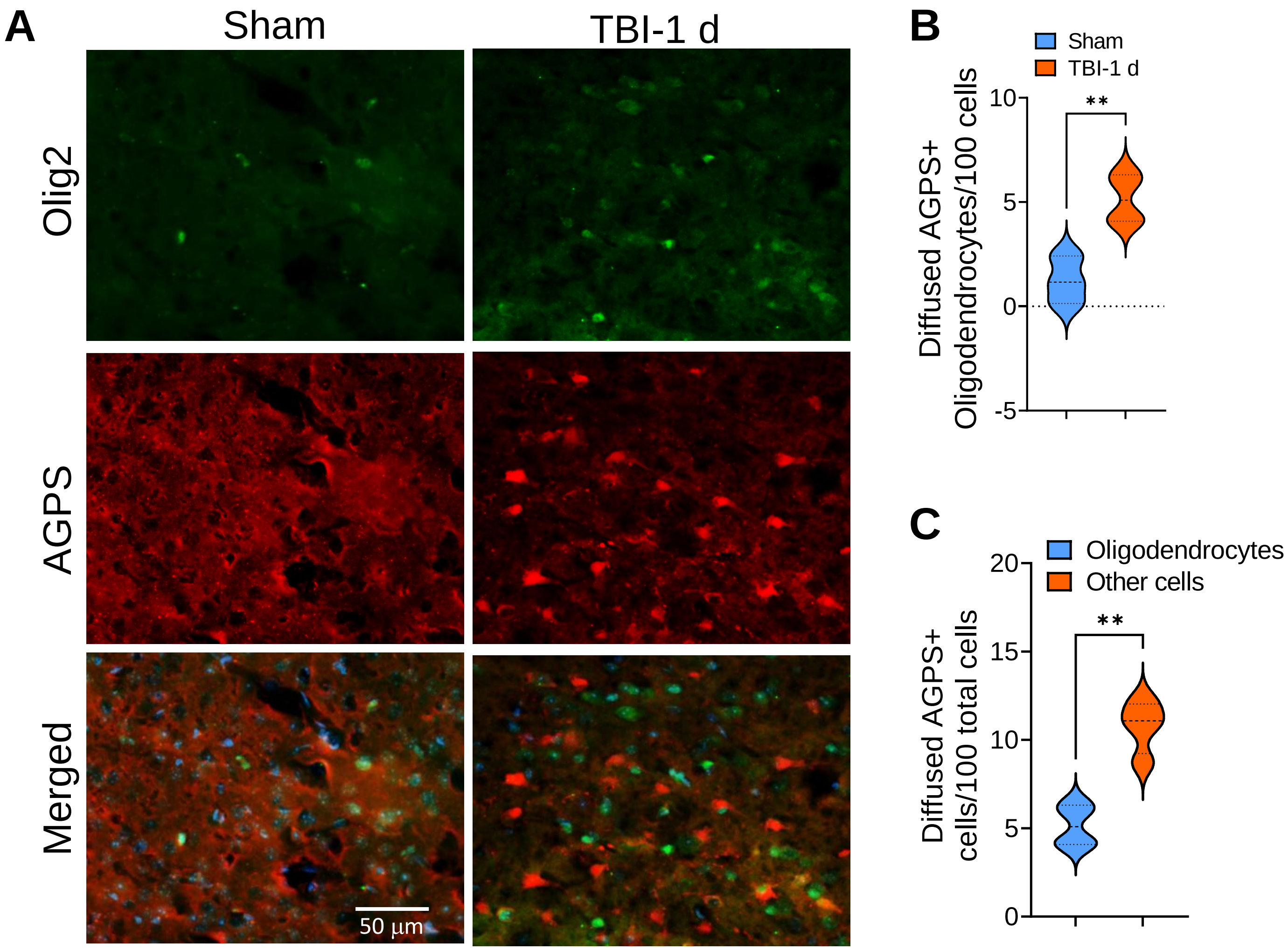

Supplemental figure 6. AGPS predominantly accumulates in other cells than oligodendrocytes after TBI. (A) 20 X images of sham and TBI mouse brain sections stained with AGPS and Olig 2 antibody. **(B)** Quantification of oligodendrocytes with diffused AGPS in sham and TBI mouse cortices and **(C)** comparative assessment of oligodendrocytes with diffused AGPS with other cell types in TBI mouse brains sections.

Figure –S7

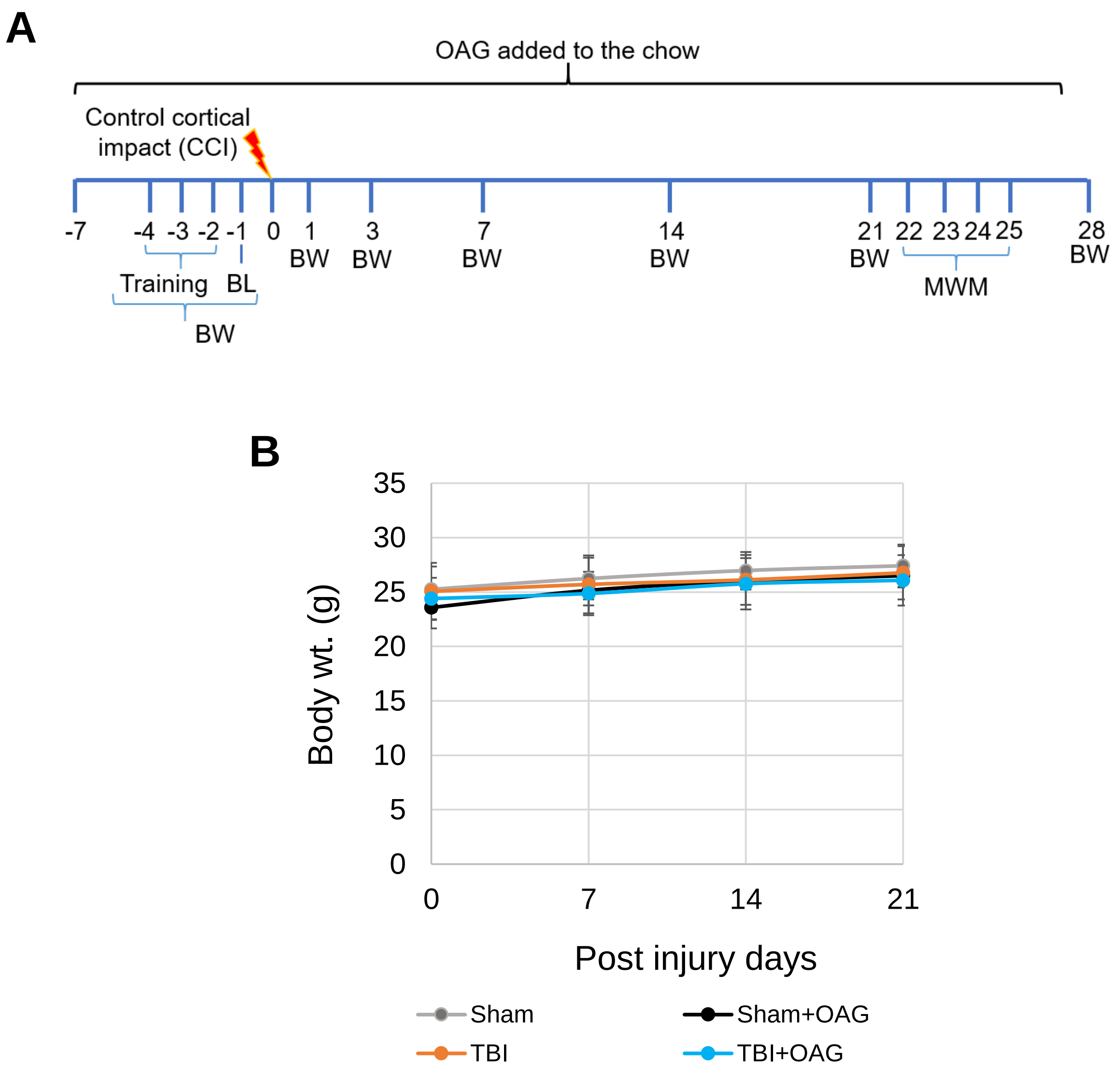

Supplemental figure 7. (A) Schematic diagram showing OAG-treatment plan. **(B)** Time-dependent changes in body weights of sham and TBI mice treated with or without OAG.

Figure –S8

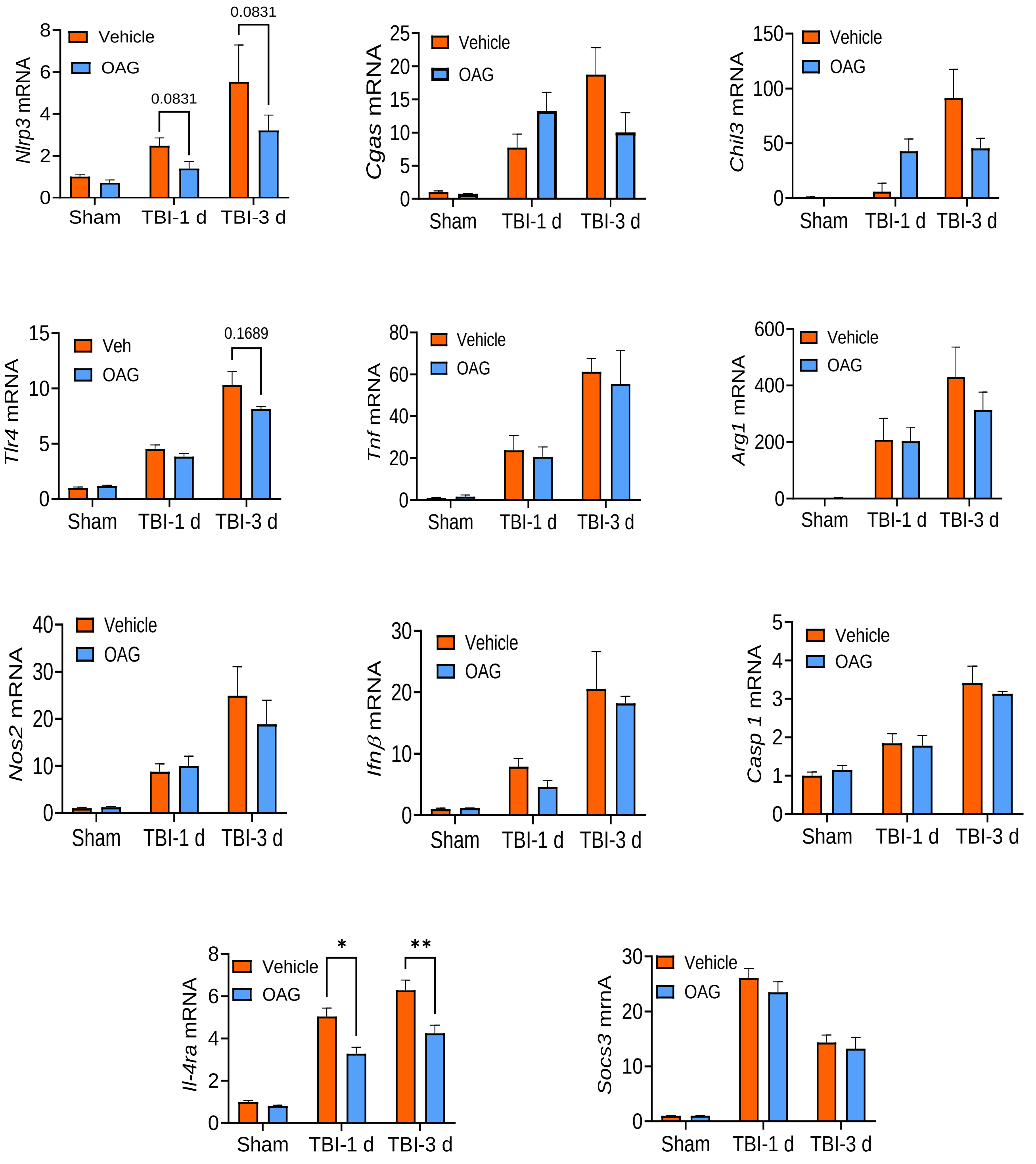

Supplemental figure 8. Relative mRNA levels (normalized to *Gapdh* mRNA) of different inflammatory markers in the cortices of sham and TBI mice (PID 1 and 3). Data=Mean ± SEM; n=4 (sham) – 6 (TBI). **P<0.01, Two-way ANOVA.
